# Causal associations between modifiable risk factors and pancreatitis: A comprehensive Mendelian randomization study

**DOI:** 10.3389/fimmu.2023.1091780

**Published:** 2023-03-14

**Authors:** Xiaotong Mao, Shenghan Mao, Hongxin Sun, Fuquan Huang, Yuanchen Wang, Deyu Zhang, Qiwen Wang, Zhaoshen Li, Wenbin Zou, Zhuan Liao

**Affiliations:** ^1^ Department of Gastroenterology, Changhai Hospital, Navy Medical University, Shanghai, China; ^2^ Shanghai Institute of Pancreatic Diseases, Shanghai, China

**Keywords:** pancreatitis, alcohol, modifiable risk factors, Mendelian randomization, lifestyle

## Abstract

**Background:**

The pathogenesis of pancreatitis involves diverse environmental risk factors, some of which have not yet been clearly elucidated. This study systematically investigated the causal effects of genetically predicted modifiable risk factors on pancreatitis using the Mendelian randomization (MR) approach.

**Methods:**

Genetic variants associated with 30 exposure factors were obtained from genome-wide association studies. Summary-level statistical data for acute pancreatitis (AP), chronic pancreatitis (CP), alcohol-induced AP (AAP) and alcohol-induced CP (ACP) were obtained from FinnGen consortia. Univariable and multivariable MR analyses were performed to identify causal risk factors for pancreatitis.

**Results:**

Genetic predisposition to smoking (OR = 1.314, *P* = 0.021), cholelithiasis (OR = 1.365, *P* = 1.307E-19) and inflammatory bowel disease (IBD) (OR = 1.063, *P* = 0.008) as well as higher triglycerides (OR = 1.189, *P* = 0.016), body mass index (BMI) (OR = 1.335, *P* = 3.077E-04), whole body fat mass (OR = 1.291, *P* = 0.004) and waist circumference (OR = 1.466, *P* = 0.011) were associated with increased risk of AP. The effect of obesity traits on AP was attenuated after correcting for cholelithiasis. Genetically-driven smoking (OR = 1.595, *P* = 0.005), alcohol consumption (OR = 3.142, *P* = 0.020), cholelithiasis (OR = 1.180, *P* = 0.001), autoimmune diseases (OR = 1.123, *P* = 0.008), IBD (OR = 1.066, *P* = 0.042), type 2 diabetes (OR = 1.121, *P* = 0.029), and higher serum calcium (OR = 1.933, *P* = 0.018), triglycerides (OR = 1.222, *P* = 0.021) and waist-to-hip ratio (OR = 1.632, *P* = 0.023) increased the risk of CP. Cholelithiasis, triglycerides and the waist-to-hip ratio remained significant predictors in the multivariable MR. Genetically predicted alcohol drinking was associated with increased risk of AAP (OR = 15.045, *P* = 0.001) and ACP (OR = 6.042, *P* = 0.014). After adjustment of alcohol drinking, genetic liability to IBD had a similar significant causal effect on AAP (OR = 1.137, *P* = 0.049), while testosterone (OR = 0.270, *P* = 0.002) a triglyceride (OR = 1.610, *P* = 0.001) and hip circumference (OR = 0.648, *P* = 0.040) were significantly associated with ACP. Genetically predicted higher education and household income levels could lower the risk of pancreatitis.

**Conclusions:**

This MR study provides evidence of complex causal associations between modifiable risk factors and pancreatitis. These findings provide new insights into potential therapeutic and prevention strategies.

## Introduction

Pancreatitis is a complex, progressive and debilitating inflammatory disease of the pancreas, the continuum of which includes clinical diagnoses of acute pancreatitis (AP), recurrent acute pancreatitis (RAP) and chronic pancreatitis (CP). AP is one of the most common gastrointestinal conditions resulting in hospital admission (1), with an estimated annual incidence of 34 cases per 100000 person-years and 1.16 deaths per 100000 person-years in high-income countries (2). Recurrent episodes of AP can eventually lead to pancreatic failure and CP. Although CP has a lower overall incidence (9.62 per 100 000 person-years) and mortality (0.09 per 100 000 person-years) than AP (2), it can result in intractable abdominal pain, endocrine/exocrine pancreatic insufficiency, impaired quality of life and reduced life expectancy (3, 4). Furthermore, CP is considered an important risk factor for developing pancreatic cancer, a highly lethal malignancy with few effective therapeutic options (5). Excessive alcohol consumption is a well-established etiological factor for both AP (~20%) and CP (40-70%) (1, 3). Some patients with pancreatitis can be diagnosed with alcohol-induced AP (AAP) or CP (ACP) based on a history of alcohol exposure.

Since inflammatory disorders of the human pancreas tend to form a continuum, AP and CP share numerous common aetiologies. Besides excessive alcohol consumption, several important risk factors for pancreatitis, including smoking, hypertriglyceridaemia and autoimmune disease, are well established (1, 3, 4, 6, 7). Gallstone disease and hypercalcemia can significantly increase the risk of AP (1, 6), while chronic kidney disease (CKD) and celiac disease are associated with an increased risk of CP (3, 7). Inflammatory bowel disease (IBD) and systemic lupus erythematosus (SLE) seem to increase the risk of pancreatitis, although exact risk estimates are not available (8). Serum parameters, including serum amylase (9), cholesterol (10, 11) and C-reactive protein (CRP) (12), are reported as potential biomarkers for pancreatitis. Additionally, observational studies suggest that pancreatitis is associated with metabolic comorbidities, including obesity (13, 14) and type 2 diabetes (T2D) (15, 16). Although observational studies can control for known confounders through statistical techniques, the existence of unknown or unmeasured confounders could influence the results. Randomized controlled trials (RCTs) are considered a standard epidemiological design for establishing a risk factor’ direct, causal effect on disease development. However, due to cost, implementation difficulty, or ethical concerns, RCTs are not always feasible.

Mendelian randomization (MR) is an instrumental variable analysis for examining causal associations between risk factors and disease outcomes in epidemiology (17). It uses genetic variants robustly associated with a risk factor as instrumental variables (IVs) and mimics a randomized controlled setting in which all other variables except the exposure of interest are randomly and equally distributed over subgroups. Thus, MR analyses are less vulnerable to bias from confounding, reverse causation and measurement errors. As an emerging method, MR analyses are increasingly applied to explore the causal association between various risk factors and pancreatitis. Based on an analysis of participants with genetic variants associated with increased plasma triglyceride levels, Hansen et al. found that higher concentrations of triglycerides, caused by genetic variants impairing lipoprotein lipase function, increase the risk of AP (18). The study by Yuan et al. revealed the causal roles of gallstone disease, diabetes, calcium, triglycerides, smoking and alcohol consumption in AP and CP (19). More recently, Mi et al. investigated the causal associations of genetically predicted blood metabolites on pancreatitis and found that elevated triglycerides levels and reduced degree of unsaturation in fatty acids increased the risk of pancreatitis (20). Nevertheless, many other modifiable risk factors for pancreatitis, such as lifestyle factors, autoimmune diseases, serum parameters and metabolic comorbidities, have not been comprehensively studied. Better understanding and management of risk factors for pancreatitis will enable clinicians to reduce and prevent the disease.

This study explores the causal effects of 30 genetically-proxied potential risk factors on the risks of AP, CP, AAP and ACP using a two-sample MR framework. The aim of this study is to provide a comprehensive overview of putative modifiable risk factors for pancreatitis and offer novel insights into the aetiology of pancreatitis.

## Methods

### MR design

**Figure 1:**
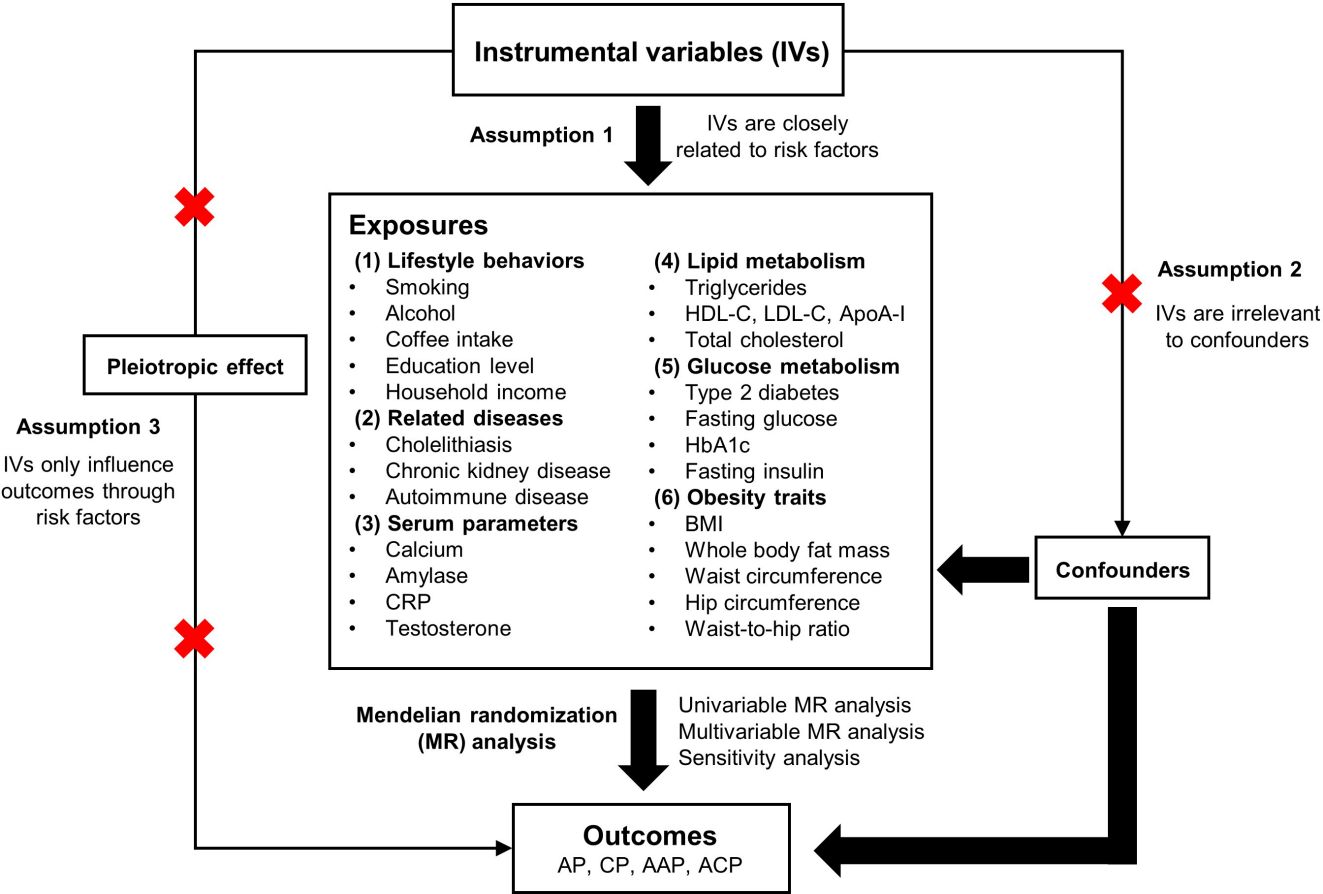
Overview of the design and methods used in this Mendelian randomization study. AP, acute pancreatitis; CP, chronic pancreatitis; AAP, alcohol-induced acute pancreatitis; ACP, alcohol-induced chronic pancreatitis; CRP, C-reactive protein; HDL-C, high-density lipoprotein cholesterol; LDL-C, low-density lipoprotein cholesterol; ApoA- I, apolipoprotein A-I; HbA1c, glycated hemoglobin; BMI, body mass index.

MR was utilised to investigate the relationships between various risk factors and different types of pancreatitis. A total of 30 primary risk factors were selected and classified into six categories: lifestyle behaviours, related diseases, serum parameters, lipid metabolism, glucose metabolism and obesity traits. Single nucleotide polymorphisms (SNPs) associated with these risk factors were used as IVs. The following three assumptions served as the foundation for the MR study: (1) the SNPs are closely related to the risk factors; (2) the SNPs are irrelevant to various confounders; (3) the SNPs only influence the outcomes through the risk factors (**Figure 1**). The datasets used in this study are available from public databases and received ethical approval before implementation. This study, therefore, did not require additional ethical approval.

### Selection of genetic instruments

Genome-wide association studies (GWASs) of individuals of European ancestry were selected as data sources for genetic instruments associated with the 30 risk factors. Genetic instruments of smoking initiation, cigarettes per day and alcoholic drinks per week were extracted from the GSCAN (GWAS and Sequencing Consortium of Alcohol and Nicotine use) consortium (21). GWAS summary statistics for coffee intake and household income were obtained from the MRC-IEU (MRC Integrative Epidemiology Unit) consortium. IVs for education level were selected from SSGAC (Social Science Genetic Association Consortium) (22). GWAS summary statistics for cholelithiasis and autoimmune diseases were obtained from the FinnGen consortium (23). IVs for IBD were extracted from IIBDGC (The International Inflammatory Bowel Disease Genetics Consortium) (24). The UK Biobank study was used as the data source for GWAS summary statistics for lipid metabolism traits including testosterone, triglycerides, high-density lipoprotein cholesterol (HDL-C), low-density lipoprotein cholesterol (LDL-C), apolipoprotein A-I and total cholesterol (25). Genetic instruments for body mass index (BMI) and whole body fat mass were selected from Neale Lab (http://www.nealelab.is/uk-biobank). GWAS summary statistics for hip circumference, waist circumference and waist-to-hip ratio were extracted from the GIANT (Genetic Investigation of ANthropometric Traits) consortium (26). For CKD (27), celiac disease (28), SLE (29), serum calcium (30), serum amylase (31), CRP (32), T2D (33), fasting glucose, HbA1c and fasting insulin (34), IVs were selected from associated GWAS studies. SNPs at the genome-wide significance level (*P* < 5×10^8^) were extracted and those with a considerable physical distance (≥ 10,000 kb) and less probability of linkage disequilibrium (R^2^ < 0.01) were included.

### GWAS summary statistics for pancreatitis cohorts

GWAS summary statistics for AP, CP, AAP and ACP were obtained from the FinnGen consortium. The R5 release of the FinnGen consortium data was used (23); this data set contains 3,022 cases and 195,144 controls for AP (https://risteys.finngen.fi/endpoints/K11_ACUTPANC), 1,737 cases and 195,144 controls for CP (https://risteys.finngen.fi/phenocode/K11_CHRONPANC), 457 cases and 218,335 controls for AAP (https://risteys.finngen.fi/phenocode/ALCOPANCACU) and 977 cases and 217,815 controls for ACP (https://risteys.finngen.fi/phenocode/ALCOPANCCHRON). All selected GWASs from the FinnGen consortium obtained ethical approval from the FinnGen Steering Committee and individuals provided informed consent.

### Evaluation of the strength of the genetic instruments

The F-statistic was used to assess the genetic instrument strength. F statistics (F = beta^2^/se^2^) were calculated for each SNP and a general F statistic was calculated for all SNPs for the corresponding exposure. F > 10 was considered to be sufficient strength. All F statistics were over 10.

### Univariable MR analyses

The random-effect inverse-variance weighted (IVW) method was utilised as the primary analysis to estimate the association between genetic liability to modifiable risk factors and the risk of pancreatitis. Given that the analysis is sensitive to outliers and horizontal pleiotropy, three sensitivity analyses, including the weighted median, MR-Egger and MR-PRESSO methods, were used to examine the consistency of the results. The weighted median model can produce unbiased estimates under the precondition that at least 50% of the selected IVs are valid (35). MR-Egger regression was used to obtain cogent causal estimates under the influence of pleiotropy (36). The MR-PRESSO method was performed to identify outlier SNPs due to the existing pleiotropy; causal effect estimates were obtained with the IVW approach after removing these outliers (37). The MR-PRESSO and Cochrane’s Q statistics were used to evaluate pleiotropy and heterogeneity, respectively.

### Multivariable MR analyses

Considering that gallstone disease (45%) is the most frequent cause of AP (1),multivariable MR analysis was undertaken with adjustment for genetically predicted cholelithiasis to assess potential mediating effects of cholelithiasis on AP risk. As alcohol consumption (40-70%) and smoking (~60%) are common aetiological risk factors for CP (3), a multivariable MR analysis with adjustment for genetically predicted alcohol consumption and genetic liability was conducted to reduce their potential pleiotropy. Furthermore, associations between the genetically predicted risk factors and alcohol-induced pancreatitis were assessed using multivariable MR analyses after adjustment for genetic liability to alcohol consumption.

The results are reported as odds ratios (OR) with corresponding 95% confidence intervals (CIs). A Bonferroni-corrected significance level of *P* < 1.67×10^-3^ (0.05/30) was used and *P* values ranging from 1.67×10^−3^ to 0.05 were classified as suggestive causal associations. All statistical analyses were performed using R 4.2.1 (R Foundation for Statistical Computing, Vienna, Austria), with the R packages “TwoSampleMR” (https://github.com/MRCIEU/TwoSampleMR) and “MRPRESSO” (https://github.com/rondolab/MR-PRESSO). Two-Sample MR analysis was performed according to the developers’ guidelines (https://mrcieu.github.io/TwoSampleMR/index.html). The data visualization was performed using the R package “forestploter” (https://github.com/adayim/forestploter).

## Results

### Baseline characteristics of the 30 candidate risk factors

Thirty potential risk factors were included in the analyses. The risk factors can be classified into six categories: lifestyle behaviours, related diseases, serum parameters, lipid metabolism, glucose metabolism and obesity traits (**Table 1**). The lifestyle behaviours include smoking, alcohol consumption, coffee consumption, education and income. The related diseases included cholelithiasis, CKD and autoimmune diseases (including celiac disease, IBD and SLE). The serum parameters include calcium, amylase, CRP and testosterone. Additionally, five traits related to lipid metabolism, four related to glucose metabolism and five pertaining to obesity traits were analysed. The number of SNPs ranged from 4 to 481. Across the 30 modifiable potential risk factors examined, the F-statistics for their respective genetic instruments were all greater than the empirical threshold of 10, suggesting no potential weak instrument bias.

**Table 1:** Characteristics of the GWAS summary data.

Exposures	GWAS ID	SNPs	Unit	Sample^*^	F	PubMed ID or Consortium
Lifestyle behaviors						
Smoking initiation	ieu-b-4877	106	SD	607291	39.80	30643251
Cigarettes per day	ieu-b-25	28	SD	337334	127.83	30643251
Alcoholic drinks per week	ieu-b-73	39	SD	335394	46.69	30643251
Coffee intake	ukb-b-5237	44	SD	428860	71.67	MRC-IEU
Education level	ieu-a-1239	481	SD	766345	46.37	30038396
Household income	ukb-b-7408	54	SD	397751	40.73	MRC-IEU
Related diseases						
Cholelithiasis	finn-b-K11_CHOLELITH	50	NA	214167	101.26	FinnGen
Chronic kidney disease	ebi-a-GCST003374	4	NA	117165	64.58	26831199
Autoimmune	finn-b-AUTOIMMUNE	73	NA	218792	112.86	FinnGen
Celiac disease	ieu-a-1058	33	logOR	24269	609.42	22057235
Inflammatory bowel disease	ieu-a-294	183	logOR	65642	103.67	26192919
Systemic lupus erythematosus	ebi-a-GCST003156	58	logOR	14267	56.75	26502338
Serum parameters						
Serum calcium	NA	7	SD	61079	304.27	24068962
Serum amylase	prot-a-89	6	NA	3301	105.74	29875488
C-reactive protein	ieu-b-35	76	NA	204402	157.98	30388399
Testosterone	ukb-d-30850_irnt	131	SD	230454	62.07	32042192
Lipid metabolism						
Triglycerides	met-d-Total_TG	100	SD	115078	130.74	UK Biobank^$^
HDL-C	met-d-HDL_C	137	SD	115078	121.61	UK Biobank^$^
LDL-C	met-d-LDL_C	71	SD	115078	164.71	UK Biobank^$^
Apolipoprotein A-I	met-d-ApoA1	108	SD	115078	118.54	UK Biobank^$^
Total cholesterol	met-d-Total_C	86	SD	115078	127.89	UK Biobank^$^
Glucose metabolism						
Type 2 diabetes	ebi-a-GCST006867	136	logOR	655666	863.97	30054458
Fasting glucose	ebi-a-GCST90002232	89	SD	200622	123.84	34059833
HbA1c	ebi-a-GCST90002244	98	SD	146806	96.23	34059833
Fasting insulin	ebi-a-GCST90002238	43	SD	151013	50.21	34059833
Obesity traits						
Body mass index	ukb-a-248	458	SD	336107	54.91	UK Biobank
Whole body fat mass	ukb-a-265	422	SD	330762	53.52	UK Biobank
Waist circumference^#^	ieu-a-67	72	SD	231353	49.58	25673412
Hip circumference^#^	ieu-a-55	90	SD	211114	56.92	25673412
Waist-to-hip ratio^#^	ieu-a-79	43	SD	210082	54.33	25673412

^*^All the samples in the GWAS datasets come from populations of European ancestry; ^#^Adjusted for body mass index; ^$^Metabolic biomarkers in the UK Biobank measured by Nightingale Health 2020; HDL-C, high-density lipoprotein cholesterol; LDL-C, low-density lipoprotein cholesterol; HbA1c, glycated hemoglobin.

### Causal effects of the modifiable risk factors on acute pancreatitis

**Figure 2:**
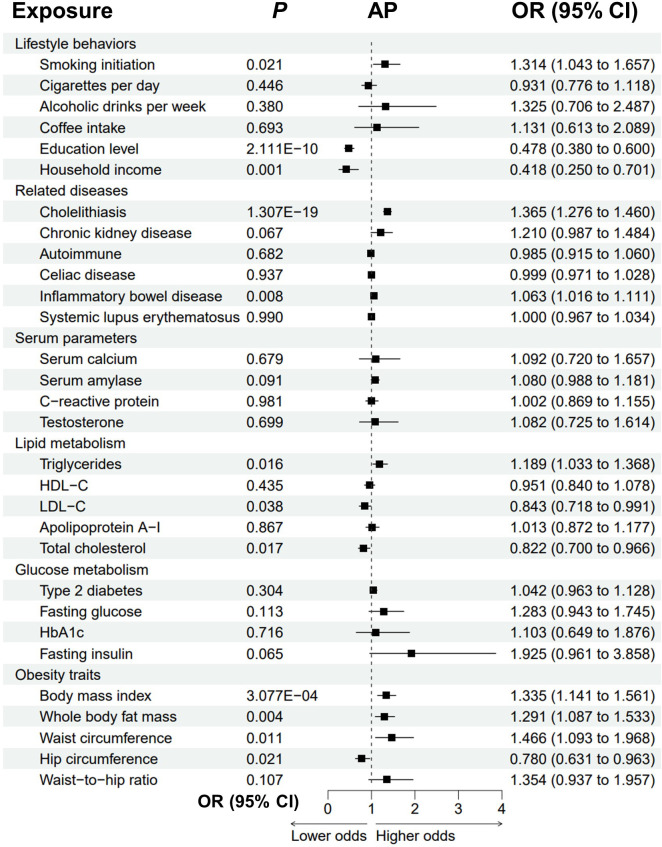
Forest plot to visualize the causal effect of modifiable risk factors on AP using the inverse variance-weighted method. AP, acute pancreatitis; OR, odds ratio; CI, confidence interval; HDL-C, high-density lipoprotein cholesterol; LDL-C, low-density lipoprotein cholesterol; HbA1c, glycated hemoglobin.

The univariable MR analyses revealed that genetically predicted cholelithiasis (OR = 1.365, *P* = 1.307E-19) and higher BMI (OR = 1.335, *P* = 3.077E-04) were significantly associated with an increased risk of AP (**Figure 2**; **Supplementary Figure 1**; **Supplementary Table 1**). Genetically predicted smoking initiation, IBD, higher triglycerides, whole body fat mass, and increased waist circumference were suggestively associated with AP. The ORs were 1.314 (*P* = 0.021) for smoking initiation, 1.189 (*P* = 0.016) for triglycerides, 1.291 (*P* = 0.004) for whole body fat mass, and 1.466 (*P* = 0.011) for waist circumference. Possible pleiotropy and heterogeneity were observed for whole body fat mass (*P*_pleiotropy_ < 0.001; *P*_heterogeneity_ = 0.003). Thus, MRPRESSO analysis was performed after removing the outliers. The relationship remained stable in the MRPRESSO-corrected results (*P* = 0.011). There was a potential association between genetically predicted IBD and increased risk of AP (OR = 1.063, *P* = 0.008). Notably, higher education and household income level were significantly associated with a reduced risk of AP. The odds of AP decreased with increas ing education level (OR = 0.478, *P* = 2.111E-10), household income (OR = 0.418, *P* = 0.001), LDL-C (OR = 0.843, *P* = 0.038), total cholesterol (OR = 0.822, *P* = 0.017) and hip circumference (OR = 0.780, *P* = 0.021). A possible pleiotropy and heterogeneity were observed for LDL-C (*P*_pleiotropy_ = 0.006; *P*_heterogeneity_ = 0.004) and total cholesterol (*P*_pleiotropy_ = 0.026; *P*_heterogeneity_ = 0.037), and these relationships remained significant in the MR-PRESSO-corrected results.

### Causal effects of the modifiable risk factors on chronic pancreatitis

**Figure 3:**
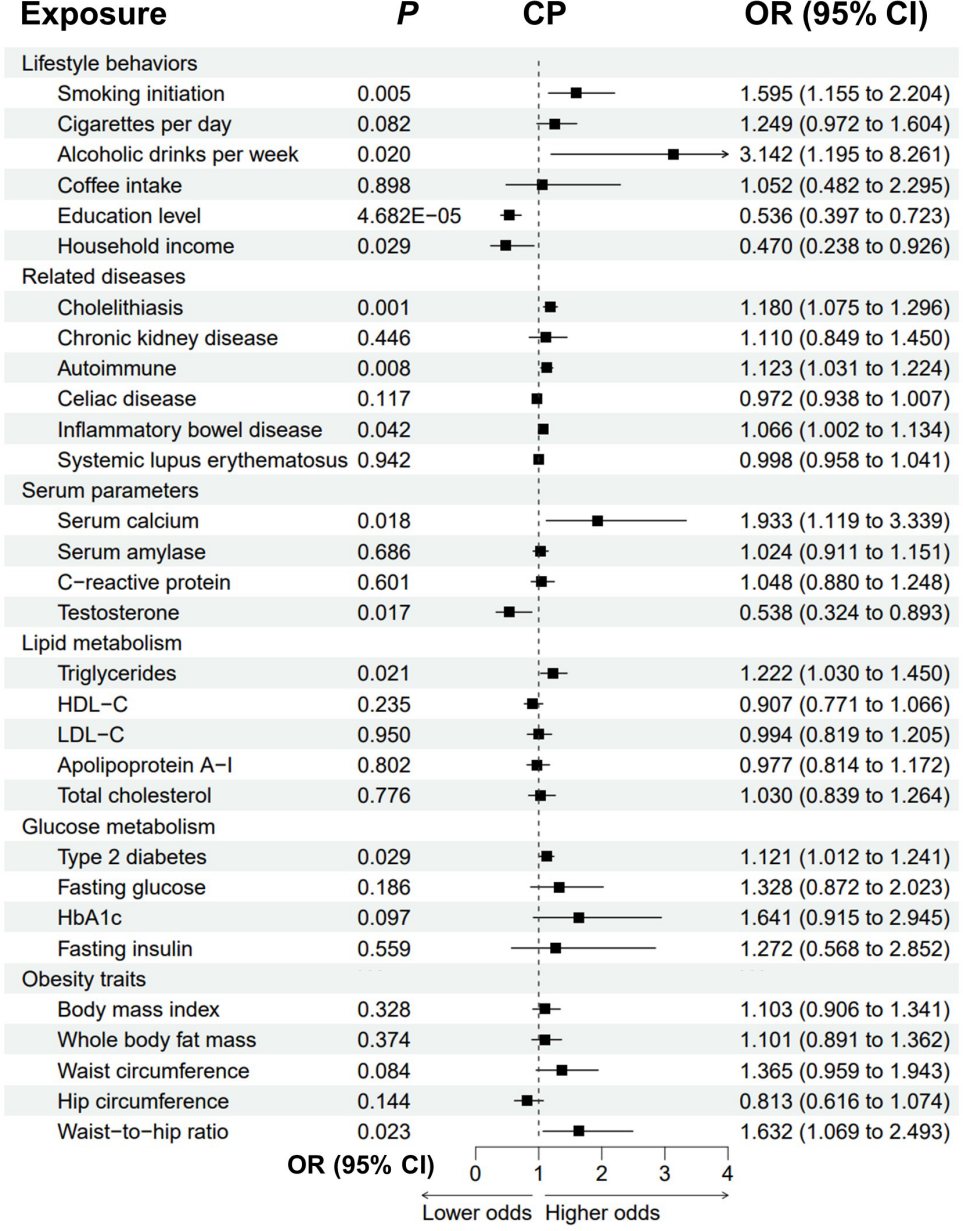
Forest plot to visualize the causal effect of modifiable risk factors on CP using the inverse variance-weighted method. CP, chronic pancreatitis; OR, odds ratio; CI, confidence interval; HDL-C, high-density lipoprotein cholesterol; LDL-C, low-density lipoprotein cholesterol; HbA1c, glycated hemoglobin.

Genetically predicted cholelithiasis was significantly associated with an increased risk of CP (OR = 1.180, *P* = 0.001), while genetically predicted smoking initiation, alcohol consumption, autoimmune diseases, IBD, T2D, and higher serum calcium, triglycerides and waist-to-hip ratio were suggestively associated with CP (**Figure 3**; **Supplementary Figure 2**; **Supplementary Table 2**). The odds of CP increased with increasing smoking initiation (OR = 1.595, *P* = 0.005), alcoholic drinks per week (OR = 3.142, *P* = 0.020), serum calcium (OR = 1.933, *P* = 0.018), triglycerides (OR = 1.222, *P* = 0.021) and waist-to-hip ratio (OR = 1.632, *P* = 0.023). Genetically predicted autoimmune diseases, IBD and T2D were suggestively associated with an increased risk of CP (autoimmune: OR = 1.123, *P* = 0.008; IBD: OR = 1.066, *P* = 0.042; T2D: OR = 1.121, *P* = 0.029). We observed possible heterogeneity for alcoholic drinks per week (*P*_heterogeneity_ = 0.05) and cholelithiasis (*P*_heterogeneity_ = 0.039). Higher education level, household income and testosterone were protective for CP (education: OR = 0.536, *P* = 4.682E-05; income: OR=0.470, *P*=0.029; testosterone: OR = 0.538, *P* = 0.017). Possible pleiotropy for education level was observed (*P*_pleiotropy_ = 0.041).

### Causal effects of the modifiable risk factors on alcohol-induced pancreatitis

**Figure 4:**
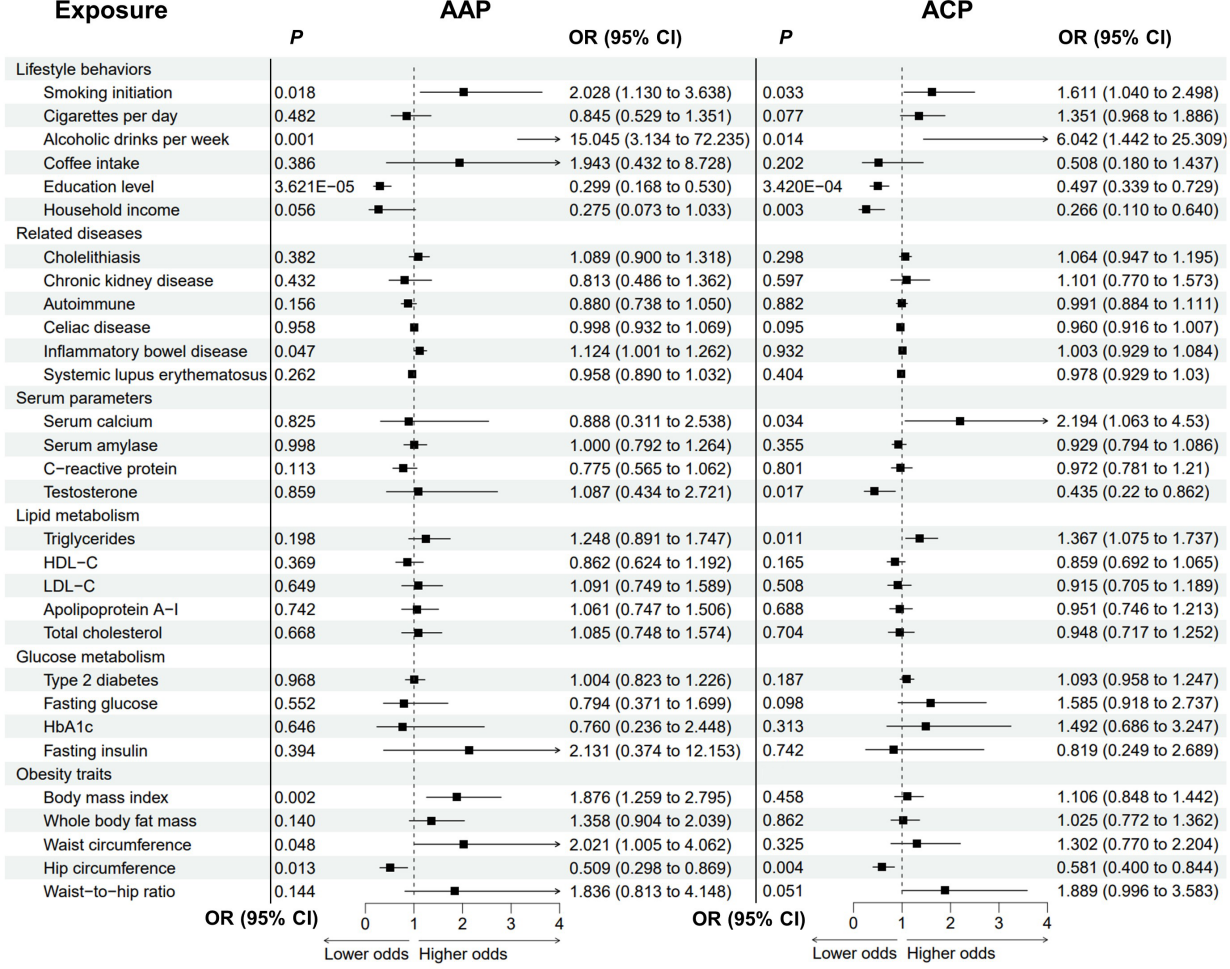
Analysis of modifiable risk factors and alcohol-induced pancreatitis by the inverse variance-weighted method. AAP, alcohol-induced acute pancreatitis; ACP, alcohol-induced chronic pancreatitis; OR, odds ratio; CI, confidence interval; HDL-C, high-density lipoprotein cholesterol; LDL-C, low-density lipoprotein cholesterol; HbA1c, glycated hemoglobin.

The causal associations between the risk factors and AAP were examined (**Figure 4**; **Supplementary Figure 3**; **Supplementary Table 3**). Notably, genetic liability to alcohol consumption was strongly associated with higher odds of AAP (OR = 15.045, *P* = 0.001). Genetic liabilities to smoking, IBD, higher BMI, and increased waist circumference were suggestively associated with an increased risk of AAP. The odds of AAP would increase with increasing smoking initiation (OR = 2.028 P = 0.018), BMI (OR = 1.876, *P* = 0.002) and waist circumference (OR = 2.021, *P* = 0.048). For BMI, there were possible pleiotropy (*P*_pleiotropy_ = 0.036) and heterogeneity (*P*_heterogeneity_ = 0.045). Genetic predisposition to IBD was associated with an increased risk of AAP (OR = 1.124, *P* = 0.047). Higher education level and increased hip circumference were protective factors for AAP (education: OR = 0.299, *P* = 3.621E-05; hip circumference: OR = 0.509, *P* = 0.013).

The causal effects of these candidate factors on ACP were then analysed (**Figure 4**; **Supplementary Figure 4**; **Supplementary Table 4**). Genetic predisposition to alcohol drinking, smoking initiation, and higher serum triglycerides and calcium suggestively correlated with ACP. The ORs were 6.042 (*P* = 0.014) for alcohol drinking, 1.611 (*P* = 0.033) for smoking initiation, 1.367 (*P* = 0.011) for triglycerides, and 2.194 (*P* = 0.034) for serum calcium. There were possible pleiotropy (*P*_pleiotropy_ = 0.006) and heterogeneity (*P*_heterogeneity_ = 0.004) for alcohol drinking. The relationship remained stable in the MRPRESSO-corrected results (*P* = 0.002). Higher education level was significantly associated with lower odds of ACP (OR = 0.497, *P* = 3.420E-04), while higher household income, higher testosterone level and increased hip circumference were suggestively protective of ACP (income: OR = 0.266, *P* = 0.003; testosterone: OR = 0.435, *P* = 0.017; hip circumference: OR = 0.581, *P* = 0.004).

### Multivariable MR analysis of pancreatitis

**Figure 5:**
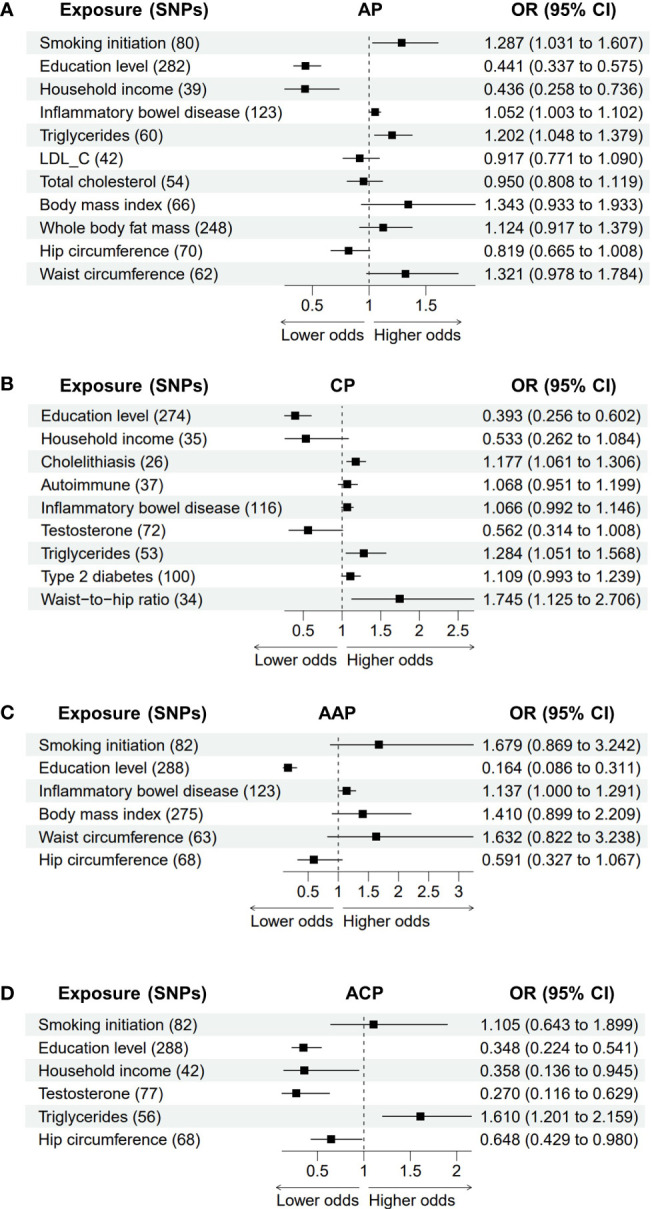
The association between adjusted modifiable risk factors and pancreatitis by multivariable Mendelian randomization. **(A)** Association between modifiable risk factors and AP after adjustment of cholelithiasis. **(B)** Association between modifiable risk factors and CP after adjustment of alcohol consumption and smoking. **(C, D)** Association between modifiable risk factors and AAP or ACP after adjustment of alcohol consumption. AP, acute pancreatitis; CP, chronic pancreatitis; AAP, alcohol-induced acute pancreatitis; ACP, alcohol-induced chronic pancreatitis; SNPs, single nucleotide polymorphisms; OR, odds ratio; CI, confidence interval; LDL-C,low-density lipoprotein cholesterol.

In the multivariable MR model, smoking (OR = 1.287, *P* = 0.026), education level (OR = 0.441, *P* = 1.78E-09), household income (OR = 0.436, *P* = 0.002), IBD (OR = 1.052, *P* = 0.036) and triglycerides (OR = 1.202, *P* = 0.009) had similar significant causal effects on AP after adjusting for genetically predicted cholelithiasis, whereas LDL_C, total cholesterol, BMI, whole body fat mass, hip circumference and waist circumference did not reach statistical significance (**Figure 5A**). This suggests that these latter associations could be affected by cholelithiasis. Adjusting for the genetic risk of alcohol consumption and smoking did not change the associations between CP and education (OR = 0.393, *P* = 1.79E-05), cholelithiasis (OR = 1.177, *P* = 0.002), triglycerides (OR = 1.284, *P* = 0.014) and the waist-to-hip ratio (OR = 0.013, *P* = 1.745). In contrast, no significant associations remained between CP and household income, autoimmune diseases, IBD, testosterone and T2D (**Figure 5B**). Finally, multivariable MR models of alcohol-induced pancreatitis were examined (**Figures 5C, D**). Education level remained a statistically significant (AAP: OR = 0.164, *P* = 3.03E-08; ACP: OR = 0.348, *P* = 2.75E-06) risk factor for alcohol-induced pancreatitis, which confirms the robustness of the results. Genetic liability to IBD had a similar significant causal effect on AAP (OR = 1.137, *P* = 0.049). Conversely, genetically predicted smoking, BMI, hip circumference and waist circumference were no longer significant risk factors for AAP in the multivariable MR model. Adjustment for alcohol consumption did not change the associations between ACP and household income (OR = 0.358, *P* = 0.038), testosterone (OR = 0.270, *P* = 0.002), triglycerides (OR = 1.610, *P* = 0.001) and hip circumference (OR = 0.648, *P* = 0.040).

## Discussion

Inflammatory diseases of the pancreas often form a continuum, with a sentinel AP event at one end of the continuum, followed by RAP and the eventual development of CP. AP is an inflammatory process with a highly variable clinical course. The causal factors implicated in AP include gallstones, alcohol abuse, hypertriglyceridemia, hypercalcemia, autoimmune diseases, medication, endoscopy, (surgical) trauma, infection and pancreatic division (1, 6). Epidemiological data have established that excessive alcohol consumption is the second leading cause of AP after gallstones (1, 6) and the most prevalent risk factor for CP (3, 7). It is also a risk factor for recurrent pancreatitis after the first AP attack and increases the risk of progression to CP (38). ACP can be diagnosed in patients who have consumed more than 80 g of alcohol on average per day for 6-12 years or have been diagnosed with alcohol addiction in the context of CP, or when symptom onset is directly associated with alcohol consumption (3). Additionally, genetic anticipation has been suggested to play an essential role in developing pancreatitis (39). The development and progression of pancreatitis are multidimensional, with interactions between genetic and environmental factors (40). In this study, the causal effects of 30 potential risk factors on pancreatitis were systematically investigated using MR analyses.

Cigarette smoking and alcohol use are two well-recognized lifestyle risk factors for pancreatitis (1, 3). Smoking promotes the progression from AP to RAP or CP and accelerates the development of alcohol-induced pancreatitis (3). MR analysis confirmed that smoking initiation was associated with a higher risk of pancreatitis. The effects of smoking on AAP and ACP were partially attenuated after adjusting for alcohol consumption, suggesting that this association is not robust enough in alcohol-induced pancreatitis. Alcohol exposure contributes to the initiation and progression of pancreatitis and amplifies the association between genetic risk factors and CP in a dose-dependent manner (41). The outcomes in the current study verify the causal associations between alcohol consumption and CP, AAP and ACP. In accordance with expectation, the risks of AAP and ACP due to genetically predicted alcohol consumption were higher than that of CP. However, there was no evidence of a positive association between alcohol consumption and AP. As highlighted by Yuan et al., the lack of this association could be due to the small proportion of moderate or heavy drinkers in the GWAS cohort (19). Furthermore, alcohol accounts for 40-70% of CP aetiologies, and only 20% of AP aetiologies. Thus, associations between AP and alcohol drinking could be less robust than associations with CP or alcohol-induced pancreatitis. The relationship between coffee consumption and pancreatitis is controversial. Some studies have reported that coffee reduces the risk of pancreatitis (42), while another prospective cohort study found no association between coffee intake and the risk of pancreatitis (43). Our MR analyses found no evidence of any associations between genetically predicted coffee consumption and pancreatitis risk.

Gallstone disease is the most common risk factor for AP in high-income countries (1); however, the relationship between gallstones and CP remains uncertain (44). The MR results indicated that genetically predicted cholelithiasis was strongly associated with a higher risk of AP events. Our results also suggested that genetically predicted cholelithiasis significantly increases the risk of CP, which is consistent with Yuan’s study (19). Autoimmune diseases, including celiac disease, IBD and SLE, have been reported to be associated with pancreatitis in previous studies (45–47). Pancreatic abnormalities in IBD include AP, CP, autoimmune pancreatitis, pancreatic exocrine insufficiency and asymptomatic abnormalities (48). The MR results in the current study support the causal relationship between IBD and pancreatitis. Nevertheless, no evidence was found for an association between pancreatitis and celiac disease or SLE. It is still a matter of debate whether AP is more prevalent in patients with CKD than in the general population. Two single-center studies demonstrated the prevalence of AP is higher in patients with end-stage renal disease (49, 50). In contrast, a recent large population-based study revealed that the prevalence of AP in the United States is comparable between advanced CKD and non-CKD (51). In the present study, genetic predisposition to CKD trended toward an increased risk of AP, but this association did not reach statistical significance.

Among the serum parameters, a possible association between genetically predicted physiologically higher calcium levels and CP or ACP was observed, consistent with Yuan et al.’s study (19). Hypertriglyceridemia is a well-established risk factor for both AP and CP, and this was supported by both the univariable and multivariable MR models in this study. LDL-C, HDL-C and apolipoprotein A-I were previously reported to be associated with the severity of AP (10, 11). However, the current results showed no associations between pancreatitis risk and genetically predicted HDL-C or apolipoprotein A-I. Genetically predicted higher LDL-C and total cholesterol were suggestively associated with lower odds of AP, whereas these associations were not significant after adjustment for cholelithiasis. Notably, the recent MR study by Chen et al. demonstrated that the odds of cholelithiasis would decrease with higher levels of total cholesterol and LDL-C (52), suggesting the protective effect of higher LDL-C or total cholesterol levels against AP could be affected by cholelithiasis. The null association between total cholesterol and pancreatitis was supported by a prospective cohort study conducted in Sweden (53).

Previous prospective studies suggested a positive link between T2D and the risk of AP (15, 54), which was already supported by the MR study by Yuan et al. (19). However, the current data suggests that genetic liability to T2D did not significantly increase the risk of AP. Two factors may influence this difference. First, the T2D GWAS dataset used by Yuan et al. combined data from multiethnic cohorts, while the T2D GWAS dataset processed in this study was obtained from European populations. Second, the updated data for AP was advantageous in the current study due to the much larger sample size. Further research is warranted to characterize and consolidate the relationship between T2D and AP. Interestingly, a suggestive relationship between T2D and CP was observed in the current study, but this relationship was not statistically significant after adjusting for smoking and alcohol consumption. Causal associations between pancreatitis and fasting glucose, HbA1c and fasting insulin were also not observed in the present study. Furthermore, the outcomes revealed suggestive associations between obesity traits and AP. However, these associations did not persist after adjusting for cholelithiasis, suggesting that an elevated risk of cholelithiasis due to obesity traits may explain this relationship (52).

Some protective factors for pancreatitis were identified in the present MR study. It is a pleasant surprise to find that a genetic liability to higher education levels significantly decreased the risk of pancreatitis in both the univariable and multivariable models. Genetically predicted household income was also associated with lower AP, CP and ACP risks. Higher education and household income may modulate pancreatitis risk by affecting multiple pathways, including individuals’ health behaviours, living environments and lifestyles. Additionally, there was a suggestive association between higher testosterone and reduced risks of CP and ACP. The potential relationship between testosterone and ACP remained significant after adjustment for alcohol consumption, which may explain previous findings indicating that female patients develop alcoholic pancreatitis at younger ages, with shorter durations, and under smaller cumulative amounts of alcohol consumption than male patients (55). Notably, testosterone has a protective effect on pancreatic beta cells, while testosterone deficiency may contribute to the development of metabolic syndrome in men (56). Furthermore, the current results suggest that bigger hip circumference was associated with reduced risks of AP, AAP and ACP, and this association persisted in the ACP cohort after adjusting for alcohol consumption.

The present study has several strengths related to the data sources and research design. First, the MR design enabled estimating the causal links between two complex heritable traits, which avoids the biases inherent in conventional observational epidemiological studies. Multiple sensitivity analyses were performed to confirm the plausibility of the instrumental variable assumptions and interpreted the results after considering horizontal pleiotropy and outliers. Second, this study has systematically analysed the largest number of modifiable causal risk factors for pancreatitis. Third, GWAS data used in this study were primarily derived from participants of European ancestry, which could reduce population stratification bias. Aside from cholelithiasis and autoimmune diseases, this study avoided sample overlap between most exposure types and outcomes, thereby keeping the type 1 error rate as low as possible. Nonetheless, some limitations of the current study also need to be considered. First, as with all MR studies, it is difficult to confirm a lack of bias due to horizontal pleiotropy. Thus, MR-Egger regression and the MR-PRESSO global test were used to detect widespread horizontal pleiotropy (36, 37). Importantly, the results of this study remained robust after the removal of outlier variants detected by the MR-PRESSO outlier test. Second, the sample size for ACP was relatively small, which could limit the statistical power to detect genuine causal relationships. Third, this study is based on individuals of European ancestry. Given the genetic differences between the races, replication studies in other populations will ensure the generalisability of the current findings.

In conclusion, the present MR study systematically elucidated the causal associations between different types of pancreatitis and various lifestyle factors, related diseases, serum parameters, lipid metabolism, glucose metabolism and obesity. This work provides a better understanding of the risk factors for the occurrence and development of pancreatitis and may inform more targeted prevention and treatment strategies.

## Data availability statement

The datasets presented in this study can be found in online repositories. The names of the repository/repositories and accession number(s) can be found in the article/**Supplementary Material**.

## Author contributions

ZhuL and WZ conceived, designed, and supervised the project. XM, SM, HS, FH, YW and DZ collected data. XM and SM performed statistical analyses. XM and SM wrote the first draft with inputs from QW, ZhaL and WZ. All authors reviewed, revised, and approved the manuscript.
