# Supplementary material for: Causal associations between modifiable risk factors and pancreatitis: A comprehensive Mendelian randomization study
Source: Front Immunol. 2023 Mar 14;14:1091780. doi: 10.3389/fimmu.2023.1091780 (PMC10043332; doi:10.3389/fimmu.2023.1091780)
Supplement: Supplementary file 1 [file DataSheet_1.pdf]

Supplementary Material

Figure legends

Supplementary Figure 1. Scatter plot of the causal relationships between modifiable risk factors and AP using different MR methods. (A) Causal estimates for smoking initiation on AP; (B) Causal estimates for education level on AP; (C) Causal estimates for household income on AP; (D) Causal estimates for cholelithiasis on AP; (E) Causal estimates for inflammatory bowel disease on AP; (F) Causal estimates for triglycerides on AP; (G) Causal estimates for LDL-C on AP; (H) Causal estimates for total cholesterol on AP; (I) Causal estimates for body mass index on AP; (J) Causal estimates for whole body fat mass on AP; (K) Causal estimates for waist circumference on AP; (L) Causal estimates for hip circumference on AP. The slopes of the line represent the causal association for different methods. The light blue line represents the Inverse variance weighted estimate, the dark blue line represents the MR Egger, the light green line represents the Simple mode estimate, the dark green line represents the weighted median estimate, and the pink line represents the weighted mode estimate.

Supplementary Figure 2. Scatter plot of the causal relationships between modifiable risk factors and CP using different MR methods. (A) Causal estimates for smoking initiation on CP; (B) Causal estimates for alcoholic drinks per week on CP (C) Causal estimates for education level on CP; (D) Causal estimates for household income on CP; (E) Causal estimates for cholelithiasis on CP; (F) Causal estimates for autoimmune on CP; (G) Causal estimates for inflammatory bowel disease on CP; (H) Causal estimates for testosterone on CP; (I) Causal estimates for triglycerides on CP; (J) Causal estimates for type 2 diabetes on CP; (K) Causal estimates for waist-to-hip ratio on CP. The slopes of the line represent the causal association for different methods. The light blue line represents the Inverse variance weighted estimate, the dark blue line represents the MR Egger, the light green line represents the Simple mode estimate, the dark green line represents the weighted median estimate, and the pink line represents the weighted mode estimate.

Supplementary Figure 3. Scatter plot of the causal relationships between modifiable risk factors and AP using different MR methods. (A) Causal estimates for smoking initiation on AAP; (B) Causal estimates for alcoholic drinks per week on AAP; (C) Causal estimates for education level on AAP; (D) Causal estimates for inflammatory bowel disease on AAP; (E) Causal estimates for body mass index on AAP; (F) Causal estimates for waist circumference on AAP; (G) Causal estimates for hip circumference on AAP. The slopes of the line represent the causal association for different methods. The light blue line represents the Inverse variance weighted estimate, the dark blue line represents the MR Egger, the light green line represents the Simple mode estimate, the dark green line represents the weighted median estimate, and the pink line represents the weighted mode estimate.

Supplementary Figure 4. Scatter plot of the causal relationships between modifiable risk factors and ACP using different MR methods. (A) Causal estimates for smoking initiation on ACP; (B) Causal estimates for alcoholic drinks per week on ACP (C) Causal estimates for education level on ACP; (D) Causal estimates for household income on ACP; (E) Causal estimates for testosterone on ACP; (F) Causal estimates for triglycerides on ACP; (G) Causal estimates for hip circumference on ACP. The slopes of the line represent the causal association for different methods. The light blue line represents

the Inverse variance weighted estimate, the dark blue line represents the MR Egger, the light green line represents the Simple mode estimate, the dark green line represents the weighted median estimate, and the pink line represents the weighted mode estimate.

Supplementary Figure 1

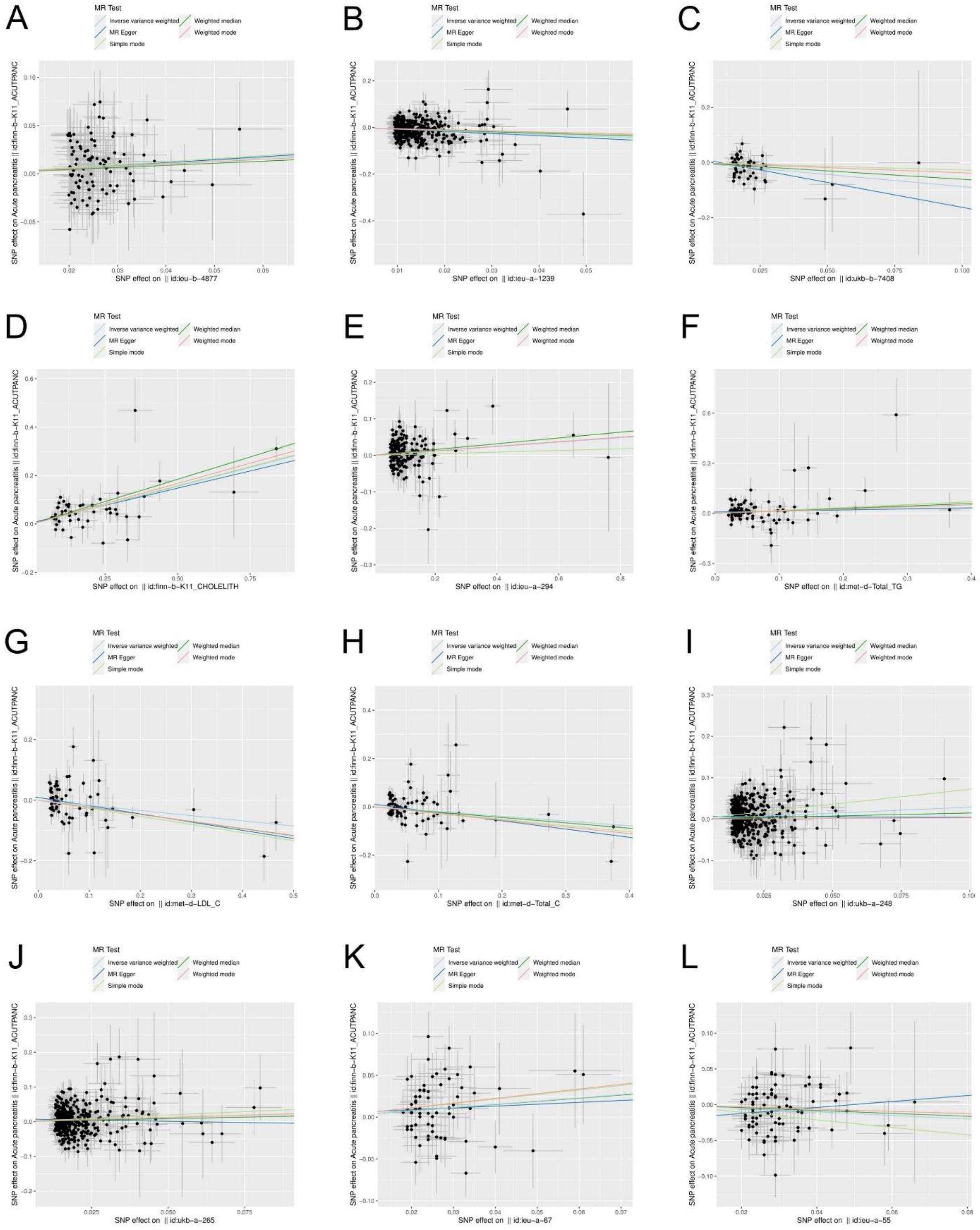

Supplementary Figure 2

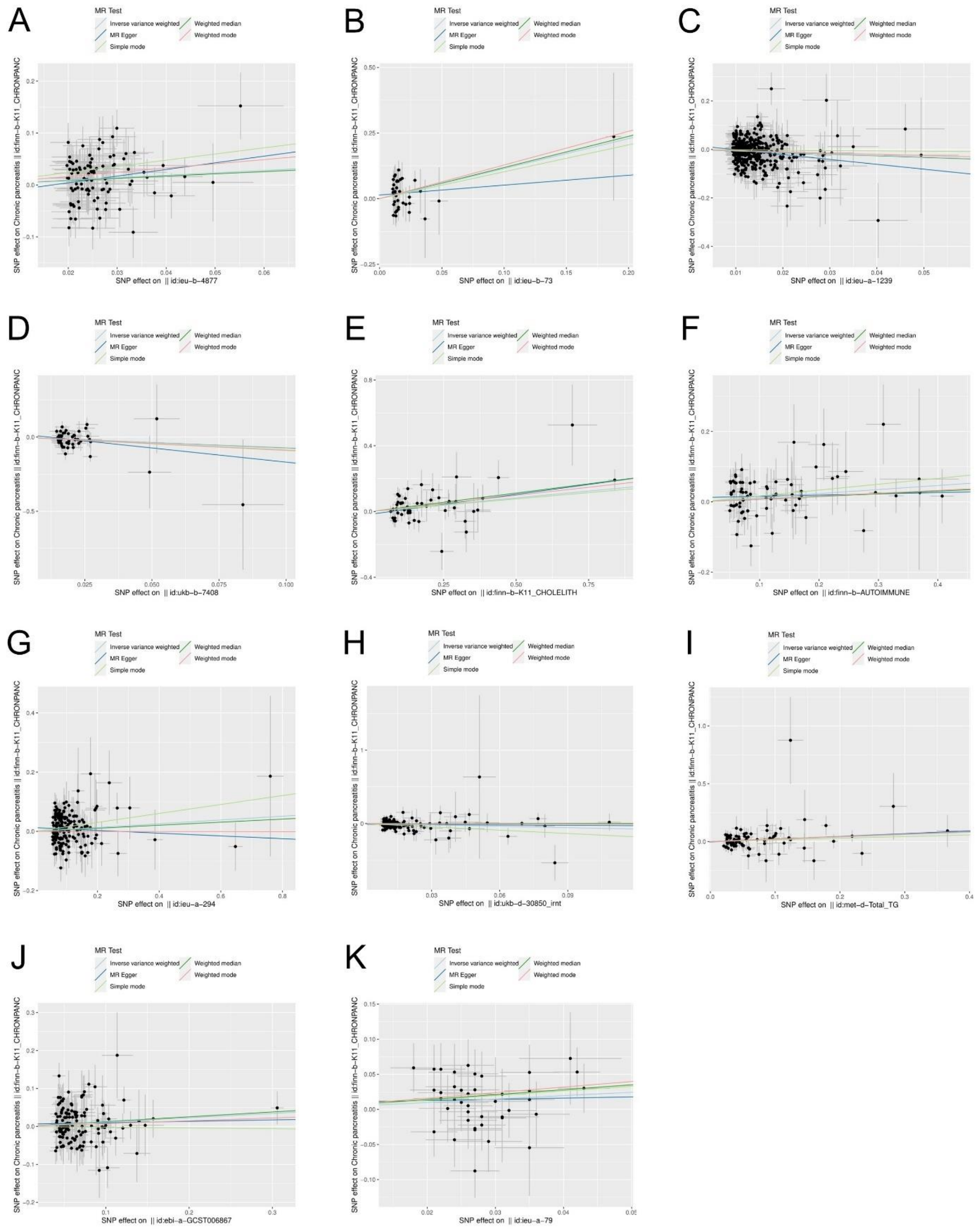

Supplementary Figure 3

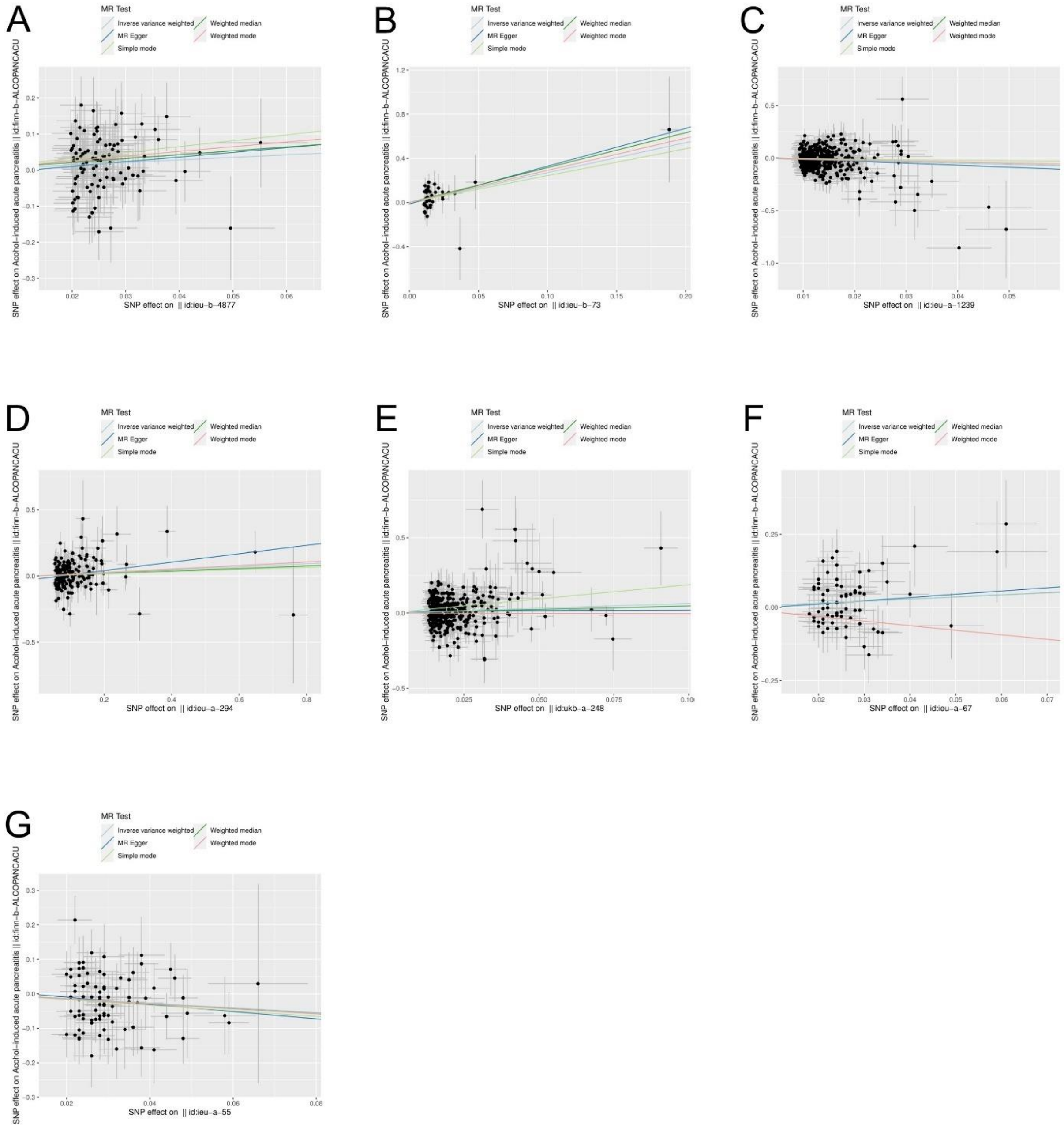

Supplementary Figure 4

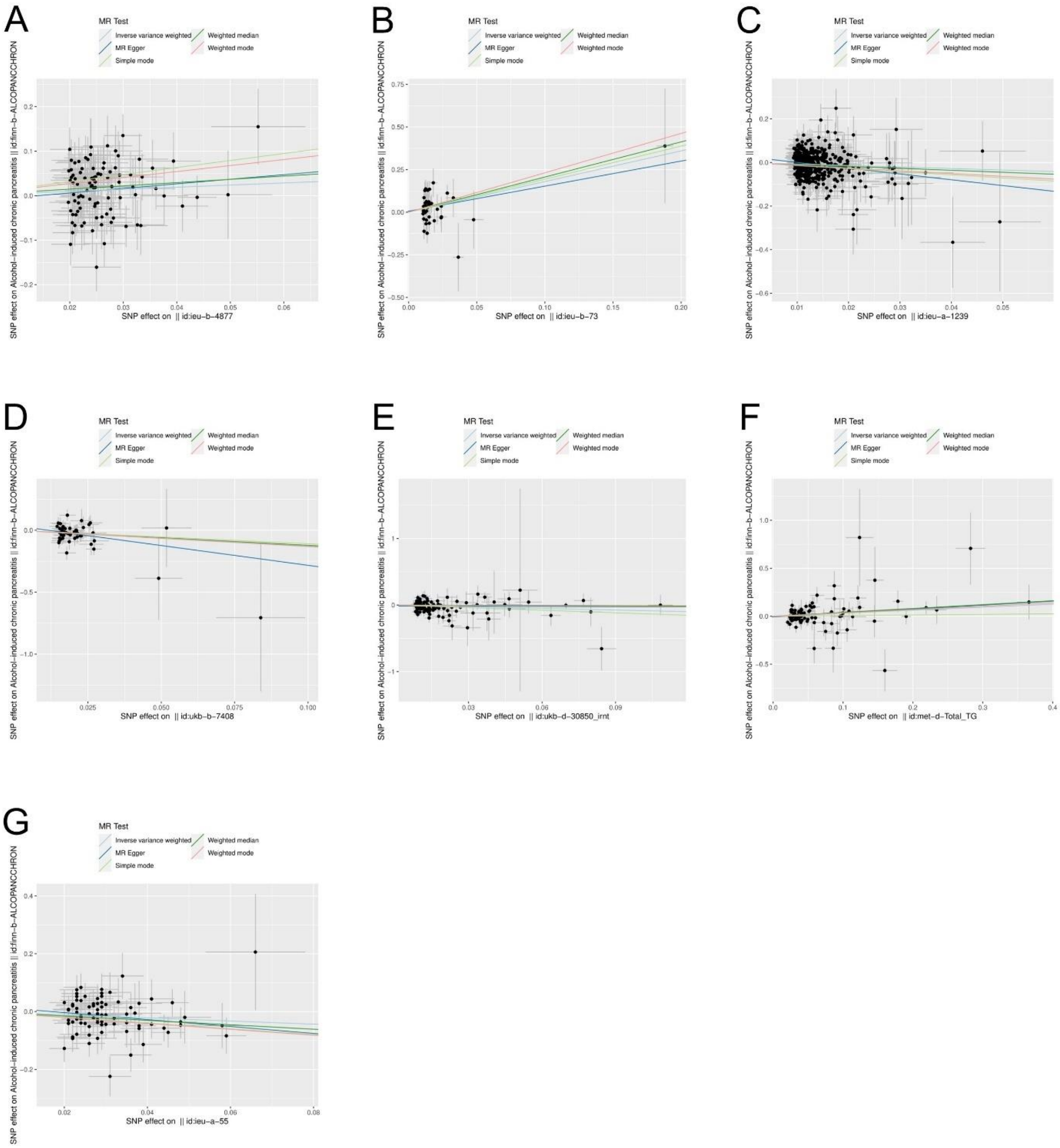

Supplenentary table 1. Modifiable risk factors for AP.

Trait	SNPs	F	IVW		WM		MR-Egger		MR-PRESSO			*P* pleiotropy	*P* heterogeneity
			OR (95% CI)	*P*	OR (95% CI)	*P*	OR (95% CI)	*P*	SNPs	OR (95% CI)	*P*		
**Lifestyle behaviors**													
Smoking initiation	96	40.10	**1.314 (1.043 to 1.657)**	**0.021**	1.242 (0.892 to 1.728)	0.199	1.362 (0.447 to 4.157)	0.588	96	**1.314 (1.043 to 1.657)**	**0.021**	0.949	0.921
Cigarettes per day	28	127.83	0.931 (0.776 to 1.118)	0.446	0.824 (0.628 to 1.082)	0.163	**0.712 (0.517 to 0.982)**	**0.048**	28	0.931 (0.776 to 1.118)	0.446	0.522	0.523
Alcoholic drinks per week	38	47.02	1.325 (0.706 to 2.487)	0.380	2.039 (0.790 to 5.262)	0.141	1.945(0.434 to 8.721)	0.391	38	1.325 (0.706 to 2.487)	0.380	0.409	0.415
Coffee intake	42	74.04	1.131 (0.613 to 2.089)	0.693	1.135 (0.488 to 2.643)	0.768	0.743 (0.211 to 2.617)	0.646	42	1.131 (0.613 to 2.089)	0.693	0.439	0.358
Education level	439	46.57	**0.478 (0.380 to 0.600)**	**0.000**	**0.543 (0.389 to 0.756)**	**0.000**	**0.387 (0.157 to 0.951)**	**0.039**	439	**0.478 (0.380 to 0.600)**	**0.000**	0.098	0.108
Household income	47	40.81	**0.418 (0.250 to 0.701)**	**0.001**	0.545 (0.273 to 1.089)	0.086	0.164 (0.011 to 2.363)	0.191	47	**0.418 (0.250 to 0.701)**	**0.001**	0.054	0.137
**Related diseases**													
Cholelithiasis	50	101.26	**1.365 (1.276 to 1.460)**	**0.000**	**1.446 (1.312 to 1.594)**	**0.000**	**1.329 (1.186 to 1.489)**	**0.000**	50	**1.365 (1.276 to 1.460)**	**0.000**	0.190	0.144
Chronic kidney disease	4	64.58	1.210 (0.987 to 1.484)	0.067	1.210 (0.951 to 1.540)	0.121	1.892 (0.954 to 3.752)	0.209	4	1.210 (0.987 to 1.484)	0.067	0.530	0.529
Autoimmune	71	115.04	0.985 (0.915 to 1.060)	0.682	0.958 (0.862 to 1.066)	0.432	0.878 (0.775 to 0.995)	0.045	71	0.985 (0.915 to 1.060)	0.682	0.064	0.073
Celiac disease	32	525.66	0.999 (0.971 to 1.028)	0.937	0.996 (0.954 to 1.040)	0.856	0.975 (0.930 to 1.022)	0.297	32	0.999 (0.971 to 1.028)	0.937	0.355	0.346
Inflammatory bowel disease	178	101.09	**1.063 (1.016 to 1.111)**	**0.008**	**1.083 (1.009 to 1.161)**	**0.026**	1.063 (0.953 to 1.185)	0.272	178	**1.063 (1.016 to 1.111)**	**0.008**	0.397	0.402
Systemic lupus erythematosus	48	61.49	1.000 (0.967 to 1.034)	0.990	1.001 (0.956 to 1.048)	0.961	0.976 (0.906 to 1.052)	0.530	48	1.000 (0.967 to 1.034)	0.990	0.121	0.099
**Serum parameters**													
Serum calcium	7	304.27	1.092 (0.720 to 1.657)	0.679	1.312 (0.806 to 2.138)	0.275	1.626 (0.752 to 3.516)	0.272	7	1.092 (0.720 to 1.657)	0.679	0.703	0.807
Serum amylase	5	105.41	1.080 (0.988 to 1.181)	0.091	1.085 (0.985 to 1.196)	0.097	1.108 (0.815 to 1.506)	0.561	5	1.080 (0.988 to 1.181)	0.091	0.952	0.932
C-reactive protein	71	163.96	1.002 (0.869 to 1.155)	0.981	0.999 (0.804 to 1.241)	0.990	1.055 (0.851 to 1.310)	0.626	71	1.002 (0.869 to 1.155)	0.981	0.051	0.051
Testosterone	117	60.05	1.082 (0.725 to 1.614)	0.699	**1.908 (1.024 to 3.556)**	**0.042**	**2.172 (1.102 to 4.281)**	**0.027**	117	1.082 (0.725 to 1.614)	0.699	0.062	0.064
**Lipid metabolism**													
Triglycerides	85	130.38	**1.189 (1.033 to 1.368)**	**0.016**	1.160 (0.933 to 1.441)	0.181	1.065 (0.845 to 1.342)	0.596	85	**1.189 (1.033 to 1.368)**	**0.016**	0.145	0.139
HDL-C	117	128.43	0.951 (0.840 to 1.078)	0.435	1.122 (0.914 to 1.377)	0.271	1.030 (0.835 to 1.272)	0.781	117	0.951 (0.840 to 1.078)	0.435	0.351	0.400
LDL-C	65	172.44	**0.843 (0.718 to 0.991)**	**0.038**	**0.791 (0.647 to 0.968)**	**0.023**	**0.763 (0.602 to 0.967)**	**0.029**	61	**0.839 (0.727 to 0.968)**	**0.016**	0.006	0.004
Apolipoprotein A-I	92	124.37	1.013 (0.872 to 1.177)	0.867	**1.263 (1.011 to 1.577)**	**0.040**	1.249 (0.962 to 1.622)	0.099	92	1.013 (0.872 to 1.177)	0.867	0.053	0.079
Total cholesterol	73	139.04	**0.822 (0.700 to 0.966)**	**0.017**	0.802 (0.643 to 1.001)	0.051	**0.711 (0.549 to 0.919)**	**0.011**	69	**0.826 (0.715 to 0.954)**	**0.009**	0.026	0.037
**Glucose metabolism**													
Type 2 diabetes	131	870.63	1.042 (0.963 to 1.128)	0.304	1.034 (0.905 to 1.182)	0.622	1.046 (0.865 to 1.263)	0.645	131	1.042 (0.963 to 1.128)	0.304	0.241	0.205
Fasting glucose	84	125.65	1.283 (0.943 to 1.745)	0.113	1.077 (0.669 to 1.733)	0.760	1.020 (0.584 to 1.781)	0.944	84	1.283 (0.943 to 1.745)	0.113	0.408	0.374
HbA1c	87	102.51	1.103 (0.649 to 1.876)	0.716	1.494 (0.712 to 3.137)	0.288	1.441 (0.508 to 4.086)	0.494	82	1.326 (0.842 to 2.088)	0.223	0.017	0.006
Fasting insulin	42	50.67	1.925 (0.961 to 3.858)	0.065	1.737 (0.723 to 4.169)	0.217	0.345 (0.042 to 2.816)	0.327	42	1.925 (0.961 to 3.858)	0.065	0.068	0.068

**Obesity traits**													
Body mass index	414	55.44	**1.335 (1.141 to 1.561)**	**0.000**	1.161 (0.899 to 1.498)	0.253	0.976 (0.606 to 1.571)	0.919	414	**1.335 (1.141 to 1.561)**	**0.000**	0.120	0.069
Whole body fat mass	396	53.53	**1.291 (1.087 to 1.533)**	**0.004**	1.194 (0.918 to 1.553)	0.187	0.870 (0.508 to 1.490)	0.613	393	**1.242 (1.051 to 1.468)**	**0.011**	<0.001	0.003
Waist circumference	70	47.59	**1.466 (1.093 to 1.968)**	**0.011**	1.456 (0.971 to 2.183)	0.069	1.252 (0.331 to 4.732)	0.742	70	**1.466 (1.093 to 1.968)**	**0.011**	0.158	0.127
Hip circumference	87	56.09	**0.780 (0.631 to 0.963)**	**0.021**	0.817 (0.596 to 1.121)	0.210	1.520 (0.676 to 3.417)	0.314	87	**0.780 (0.631 to 0.963)**	**0.021**	0.619	0.593
Waist-to-hip ratio	41	54.36	1.354 (0.937 to 1.957)	0.107	1.335 (0.826 to 2.157)	0.238	1.235 (0.203 to 7.532)	0.820	41	1.354 (0.937 to 1.957)	0.107	0.139	0.096

SNPs: single nucleotide polymorphisms; IVW: inverse variance weighted; WM: weighted median; MR-PRESSO: MR-pleiotropy residual sum and outlier; OR: odds ratio; CI: confidence interval; HDL-C: high-density lipoprotein cholesterol; LDL-C: low-density lipoprotein cholesterol; HbA1c: glycated hemoglobin.

Supplenentary table 2. Modifiable risk factors for CP.

Trait	SNPs	F	IVW		WM		MR-Egger			MR-PRESSO		*P* pleiotropy	*P* heterogeneity
			OR (95% CI)	*P*	OR (95% CI)	*P*	OR (95% CI)	*P*	SNPs	OR (95% CI)	*P*		
**Lifestyle behaviors**													
Smoking initiation	96	40.10	**1.595 (1.155 to 2.204)**	**0.005**	1.534 (0.975 to 2.413)	0.064	3.602 (0.760 to 17.084)	0.110	96	**1.595 (1.155 to 2.204)**	**0.005**	0.198	0.181
Cigarettes per day	28	127.83	1.249 (0.972 to 1.604)	0.082	1.121 (0.787 to 1.598)	0.526	0.969 (0.629 to 1.492)	0.886	28	1.249 (0.972 to 1.604)	0.082	0.392	0.339
Alcoholic drinks per week	38	47.02	**3.142 (1.195 to 8.261)**	**0.020**	3.278 (0.899 to 11.954)	0.072	1.458 (0.146 to 14.544)	0.750	38	**3.142 (1.195 to 8.261)**	**0.020**	0.062	0.050
Coffee intake	42	74.04	1.052 (0.482 to 2.295)	0.898	1.113 (0.388 to 3.195)	0.842	1.069 (0.218 to 5.250)	0.935	42	1.052 (0.482 to 2.295)	0.898	0.996	0.993
Education level	439	46.57	**0.536 (0.397 to 0.723)**	**0.000**	**0.519 (0.342 to 0.789)**	**0.002**	**0.134 (0.041 to 0.436)**	**0.001**	436	**0.560 (0.418 to 0.750)**	**0.000**	0.041	0.094
Household income	47	40.81	**0.470 (0.238 to 0.926)**	**0.029**	0.479 (0.194 to 1.184)	0.111	0.148 (0.004 to 4.940)	0.291	47	**0.470 (0.238 to 0.926)**	**0.029**	0.125	0.133
**Related diseases**													
Cholelithiasis	50	101.26	**1.180 (1.075 to 1.296)**	**0.001**	**1.252 (1.101 to 1.424)**	**0.001**	**1.272 (1.089 to 1.486)**	**0.004**	50	**1.180 (1.075 to 1.296)**	**0.001**	0.051	0.039
Chronic kidney disease	4	64.58	1.110 (0.849 to 1.450)	0.446	1.097 (0.789 to 1.526)	0.581	1.441 (0.587 to 3.538)	0.509	4	1.110 (0.849 to 1.450)	0.446	0.645	0.548
Autoimmune	71	115.04	**1.123 (1.031 to 1.224)**	**0.008**	1.079 (0.948 to 1.228)	0.249	1.036 (0.892 to 1.203)	0.642	71	**1.123 (1.031 to 1.224)**	**0.008**	0.599	0.599
Celiac disease	32	525.66	0.972 (0.938 to 1.007)	0.117	0.973 (0.926 to 1.022)	0.274	0.987 (0.930 to 1.048)	0.671	32	0.972 (0.938 to 1.007)	0.117	0.582	0.546
Inflammatory bowel disease	178	101.09	**1.066 (1.002 to 1.134)**	**0.042**	1.053 (0.960 to 1.155)	0.273	0.955 (0.823 to 1.107)	0.540	178	**1.066 (1.002 to 1.134)**	**0.042**	0.143	0.122
Systemic lupus erythematosus	48	61.49	0.998 (0.958 to 1.041)	0.942	0.987 (0.930 to 1.047)	0.659	0.997 (0.908 to 1.093)	0.941	48	0.998 (0.958 to 1.041)	0.942	0.277	0.237
**Serum parameters**													
Serum calcium	7	304.27	**1.933 (1.119 to 3.339)**	**0.018**	**1.832 (1.015 to 3.306)**	**0.044**	1.586 (0.578 to 4.354)	0.412	7	**1.933 (1.119 to 3.339)**	**0.018**	0.891	0.897
Serum amylase	5	105.41	1.024 (0.911 to 1.151)	0.686	1.013 (0.891 to 1.152)	0.840	1.009 (0.675 to 1.508)	0.967	5	1.024 (0.911 to 1.151)	0.686	0.707	0.739
C-reactive protein	71	163.96	1.048 (0.880 to 1.248)	0.601	1.160 (0.900 to 1.495)	0.252	1.085 (0.832 to 1.414)	0.549	71	1.048 (0.880 to 1.248)	0.601	0.119	0.209
Testosterone	117	60.05	**0.538 (0.324 to 0.893)**	**0.017**	1.009 (0.425 to 2.397)	0.983	0.848 (0.353 to 2.037)	0.713	117	**0.538 (0.324 to 0.893)**	**0.017**	0.218	0.167
**Lipid metabolism**													
Triglycerides	85	130.38	**1.222 (1.030 to 1.450)**	**0.021**	1.241 (0.939 to 1.640)	0.129	1.270 (0.959 to 1.683)	0.100	85	**1.222 (1.030 to 1.450)**	**0.021**	0.818	0.748
HDL-C	117	128.43	0.907 (0.771 to 1.066)	0.235	0.866 (0.645 to 1.163)	0.339	0.866 (0.660 to 1.138)	0.304	117	0.907 (0.771 to 1.066)	0.235	0.804	0.834
LDL-C	65	172.44	0.994 (0.819 to 1.205)	0.950	0.962 (0.744 to 1.245)	0.770	0.928 (0.697 to 1.235)	0.608	65	0.994 (0.819 to 1.205)	0.950	0.077	0.070
Apolipoprotein A-I	92	124.37	0.977 (0.814 to 1.172)	0.802	0.958 (0.713 to 1.287)	0.777	1.048 (0.758 to 1.448)	0.778	92	0.977 (0.814 to 1.172)	0.802	0.284	0.372
Total cholesterol	73	139.04	1.030 (0.839 to 1.264)	0.776	0.973 (0.731 to 1.295)	0.851	0.816 (0.590 to 1.130)	0.226	73	1.030 (0.839 to 1.264)	0.776	0.072	0.083
**Glucose metabolism**													
Type 2 diabetes	131	870.63	**1.121 (1.012 to 1.241)**	**0.029**	1.135 (0.951 to 1.355)	0.160	1.039 (0.814 to 1.326)	0.762	131	**1.121 (1.012 to 1.241)**	**0.029**	0.285	0.290
Fasting glucose	84	125.65	1.328 (0.872 to 2.023)	0.186	1.262 (0.684 to 2.328)	0.457	1.826 (0.853 to 3.911)	0.125	84	1.328 (0.872 to 2.023)	0.186	0.197	0.193
HbA1c	87	102.51	1.641 (0.915 to 2.945)	0.097	1.658 (0.662 to 4.154)	0.281	2.429 (0.772 to 7.644)	0.133	87	1.641 (0.915 to 2.945)	0.097	0.470	0.473
Fasting insulin	42	50.67	1.272 (0.568 to 2.852)	0.559	1.665 (0.540 to 5.136)	0.375	0.109 (0.010 to 1.235)	0.081	42	1.272 (0.568 to 2.852)	0.559	0.382	0.376

**Obesity traits**													
Body mass index	414	55.44	1.103 (0.906 to 1.341)	0.328	1.043 (0.740 to 1.470)	0.810	0.998 (0.550 to 1.814)	0.996	414	1.103 (0.906 to 1.341)	0.328	0.418	0.472
Whole body fat mass	396	53.53	1.101 (0.891 to 1.362)	0.374	1.013 (0.721 to 1.423)	0.941	0.998 (0.512 to 1.946)	0.996	396	1.101 (0.891 to 1.362)	0.374	0.129	0.150
Waist circumference	70	47.59	1.365 (0.959 to 1.943)	0.084	1.258 (0.755 to 2.094)	0.378	1.499 (0.304 to 7.408)	0.621	70	1.365 (0.959 to 1.943)	0.084	0.519	0.473
Hip circumference	87	56.09	0.813 (0.616 to 1.074)	0.144	0.691 (0.462 to 1.033)	0.072	0.761 (0.263 to 2.203)	0.615	87	0.813 (0.616 to 1.074)	0.144	0.918	0.895
Waist-to-hip ratio	41	54.36	**1.632 (1.069 to 2.493)**	**0.023**	**2.014 (1.111 to 3.649)**	**0.021**	1.192 (0.153 to 9.278)	0.868	41	**1.632 (1.069 to 2.493)**	**0.023**	0.843	0.777

SNPs: single nucleotide polymorphisms; IVW: inverse variance weighted; WM: weighted median; MR-PRESSO: MR-pleiotropy residual sum and outlier; OR: odds ratio; CI: confidence interval; HDL-C: high-density lipoprotein cholesterol; LDL-C: low-density lipoprotein cholesterol; HbA1c: glycated hemoglobin.

Supplenentary table 3. Modifiable risk factors for AAP.

Trait	SNPs	F	IVW		WM		MR-Egger		MR-PRESSO			*P* pleiotropy	*P* heterogeneity
			OR (95% CI)	*P*	OR (95% CI)	*P*	OR (95% CI)	*P*	SNPs	OR (95% CI)	*P*		
**Lifestyle behaviors**													
Smoking initiation	96	40.10	**2.028 (1.130 to 3.638)**	**0.018**	**2.915 (1.249 to 6.803)**	**0.013**	3.643 (0.218 to 60.876)	0.370	96	**2.028 (1.130 to 3.638)**	**0.018**	0.649	0.689
Cigarettes per day	28	127.83	0.845 (0.529 to 1.351)	0.482	0.906 (0.477 to 1.719)	0.762	1.132 (0.495 to 2.589)	0.771	28	0.845 (0.529 to 1.351)	0.482	0.459	0.419
Alcoholic drinks per week	38	47.02	**15.045 (3.134 to 72.235)**	**0.001**	**23.650 (2.308 to 242.322)**	**0.008**	30.720 (0.735 to 1284.490)	0.081	38	**15.045 (3.134 to 72.235)**	**0.001**	0.584	0.558
Coffee intake	42	74.04	1.943 (0.432 to 8.728)	0.386	1.620 (0.195 to 13.462)	0.655	2.280 (0.102 to 51.012)	0.606	42	1.943 (0.432 to 8.728)	0.386	0.573	0.467
Education level	439	46.57	**0.299 (0.168 to 0.530)**	**0.000**	**0.303 (0.136 to 0.673)**	**0.003**	0.146 (0.015 to 1.399)	0.096	439	**0.299 (0.168 to 0.530)**	**0.000**	0.197	0.136
Household income	47	40.81	0.275 (0.073 to 1.033)	0.056	**0.105 (0.019 to 0.578)**	**0.010**	0.142 (0.000 to 137.739)	0.581	47	0.275 (0.073 to 1.033)	0.056	0.076	0.107
**Related diseases**													
Cholelithiasis	50	101.26	1.089 (0.900 to 1.318)	0.382	1.027 (0.795 to 1.326)	0.841	1.009 (0.734 to 1.387)	0.958	46	1.087 (0.921 to 1.283)	0.324	0.010	0.006
Chronic kidney disease	4	64.58	0.813 (0.486 to 1.362)	0.432	0.856 (0.466 to 1.571)	0.615	0.844 (0.150 to 4.756)	0.866	4	0.813 (0.486 to 1.362)	0.432	0.862	0.842
Autoimmune	71	115.04	0.880 (0.738 to 1.050)	0.156	0.784 (0.602 to 1.020)	0.070	0.762 (0.561 to 1.035)	0.086	71	0.880 (0.738 to 1.050)	0.156	0.214	0.203
Celiac disease	32	525.66	0.998 (0.932 to 1.069)	0.958	1.011 (0.918 to 1.114)	0.826	0.966 (0.861 to 1.085)	0.565	32	0.998 (0.932 to 1.069)	0.958	0.962	0.959
Inflammatory bowel disease	178	101.09	**1.124 (1.001 to 1.262)**	**0.047**	1.100 (0.919 to 1.317)	0.299	**1.375 (1.041 to 1.816)**	**0.026**	178	**1.124 (1.001 to 1.262)**	**0.047**	0.272	0.251
Systemic lupus erythematosus	48	61.49	0.958 (0.890 to 1.032)	0.262	0.927 (0.831 to 1.035)	0.179	0.968 (0.821 to 1.142)	0.701	48	0.958 (0.890 to 1.032)	0.262	0.756	0.694
**Serum parameters**													
Serum calcium	7	304.27	0.888 (0.311 to 2.538)	0.825	1.174 (0.343 to 4.017)	0.798	2.297 (0.330 to 15.983)	0.439	7	0.888 (0.311 to 2.538)	0.825	0.709	0.713
Serum amylase	5	105.41	1.000 (0.792 to 1.264)	0.998	1.044 (0.815 to 1.336)	0.735	1.698 (0.779 to 3.698)	0.275	5	1.000 (0.792 to 1.264)	0.998	0.451	0.370
C-reactive protein	71	163.96	0.775 (0.565 to 1.062)	0.113	0.750 (0.461 to 1.218)	0.244	0.807 (0.501 to 1.299)	0.380	71	0.775 (0.565 to 1.062)	0.113	0.993	0.986
Testosterone	117	60.05	1.087 (0.434 to 2.721)	0.859	2.146 (0.472 to 9.768)	0.323	2.339 (0.478 to 11.458)	0.297	117	1.087 (0.434 to 2.721)	0.859	0.748	0.842
**Lipid metabolism**													
Triglycerides	85	130.38	1.248 (0.891 to 1.747)	0.198	1.034 (0.601 to 1.780)	0.904	1.519 (0.871 to 2.648)	0.145	85	1.248 (0.891 to 1.747)	0.198	0.485	0.358
HDL-C	117	128.43	0.862 (0.624 to 1.192)	0.369	1.076 (0.607 to 1.908)	0.803	0.990 (0.573 to 1.710)	0.970	117	0.862 (0.624 to 1.192)	0.369	0.242	0.262
LDL-C	65	172.44	1.091 (0.749 to 1.589)	0.649	1.039 (0.637 to 1.696)	0.877	1.224 (0.701 to 2.136)	0.480	65	1.091 (0.749 to 1.589)	0.649	0.088	0.052
Apolipoprotein A-I	92	124.37	1.061 (0.747 to 1.506)	0.742	1.120 (0.595 to 2.110)	0.725	1.110 (0.595 to 2.071)	0.743	92	1.061 (0.747 to 1.506)	0.742	0.439	0.376
Total cholesterol	73	139.04	1.085 (0.748 to 1.574)	0.668	1.047 (0.606 to 1.810)	0.869	1.117 (0.610 to 2.043)	0.722	73	1.085 (0.748 to 1.574)	0.668	0.167	0.254
**Glucose metabolism**													
Type 2 diabetes	131	870.63	1.004 (0.823 to 1.226)	0.968	0.823 (0.592 to 1.145)	0.248	1.114 (0.692 to 1.795)	0.656	131	1.004 (0.823 to 1.226)	0.968	0.211	0.218
Fasting glucose	84	125.65	0.794 (0.371 to 1.699)	0.552	0.499 (0.146 to 1.708)	0.268	0.255 (0.064 to 1.008)	0.055	84	0.794 (0.371 to 1.699)	0.552	0.692	0.778
HbA1c	87	102.51	0.760 (0.236 to 2.448)	0.646	1.144 (0.184 to 7.120)	0.885	0.518 (0.052 to 5.169)	0.577	87	0.760 (0.236 to 2.448)	0.646	0.206	0.284

Fasting insulin	42	50.67	2.131 (0.374 to 12.153)	0.394	4.837 (0.459 to 50.922)	0.189	25.574 (0.117 to 5571.344)	0.245	42	2.131 (0.374 to 12.153)	0.394	0.093	0.079
**Obesity traits**													
Body mass index	414	55.44	**1.876 (1.259 to 2.795)**	**0.002**	1.591 (0.821 to 3.082)	0.169	1.070 (0.318 to 3.598)	0.913	413	**1.838 (1.239 to 2.725)**	**0.002**	0.036	0.045
Whole body fat mass	396	53.53	1.358 (0.904 to 2.039)	0.140	1.358 (0.707 to 2.607)	0.358	0.687 (0.192 to 2.461)	0.565	396	1.358 (0.904 to 2.039)	0.140	0.190	0.187
Waist circumference	70	47.59	**2.021 (1.005 to 4.062)**	**0.048**	2.039 (0.757 to 5.487)	0.158	3.048 (0.130 to 71.696)	0.491	70	**2.021 (1.005 to 4.062)**	**0.048**	0.331	0.346
Hip circumference	87	56.09	**0.509 (0.298 to 0.869)**	**0.013**	0.504 (0.226 to 1.122)	0.093	0.350 (0.045 to 2.748)	0.321	87	**0.509 (0.298 to 0.869)**	**0.013**	0.558	0.477
Waist-to-hip ratio	41	54.36	1.836 (0.813 to 4.148)	0.144	1.319 (0.389 to 4.467)	0.657	1.461 (0.027 to 79.151)	0.853	41	1.836 (0.813 to 4.148)	0.144	0.495	0.474

SNPs: single nucleotide polymorphisms; IVW: inverse variance weighted; WM: weighted median; MR-PRESSO: MR-pleiotropy residual sum and outlier; OR: odds ratio; CI: confidence interval; HDL-C: high-density lipoprotein cholesterol; LDL-C: low-density lipoprotein cholesterol; HbA1c: glycated hemoglobin.

Supplenentary table 4. Modifiable risk factors for ACP.

Trait	SNPs	F	IVW		WM		MR-Egger		MR-PRESSO			*P* pleiotropy	*P* heterogeneity
			OR (95% CI)	*P*	OR (95% CI)	*P*	OR (95% CI)	*P*	SNPs	OR (95% CI)	*P*		
**Lifestyle behaviors**													
Smoking initiation	96	40.10	**1.611 (1.040 to 2.498)**	**0.033**	**2.084 (1.123 to 3.870)**	**0.020**	2.798 (0.336 to 23.305)	0.344	96	**1.611 (1.040 to 2.498)**	**0.033**	0.082	0.118
Cigarettes per day	28	127.83	1.351 (0.968 to 1.886)	0.077	1.040 (0.667 to 1.622)	0.862	0.854 (0.488 to 1.495)	0.585	28	1.351 (0.968 to 1.886)	0.077	0.366	0.340
Alcoholic drinks per week	38	47.02	**6.042 (1.442 to 25.309)**	**0.014**	**7.867 (1.370 to 45.165)**	**0.021**	4.349 (0.135 to 139.812)	0.412	35	**7.332 (2.032 to 26.457)**	**0.002**	0.006	0.004
Coffee intake	42	74.04	0.508 (0.180 to 1.437)	0.202	0.642 (0.146 to 2.828)	0.558	0.372 (0.045 to 3.113)	0.367	42	0.508 (0.180 to 1.437)	0.202	0.995	0.995
Education level	439	46.57	**0.497 (0.339 to 0.729)**	**0.000**	**0.406 (0.227 to 0.728)**	**0.002**	**0.070 (0.016 to 0.317)**	**0.001**	439	**0.497 (0.339 to 0.729)**	**0.000**	0.822	0.817
Household income	47	40.81	**0.266 (0.110 to 0.640)**	**0.003**	**0.295 (0.091 to 0.953)**	**0.041**	0.041 (0.000 to 3.895)	0.176	47	**0.266 (0.110 to 0.640)**	**0.003**	0.216	0.200
**Related diseases**													
Cholelithiasis	50	101.26	1.064 (0.947 to 1.195)	0.298	**1.201 (1.015 to 1.420)**	**0.033**	1.202 (0.995 to 1.452)	0.063	50	1.064 (0.947 to 1.195)	0.298	0.124	0.130
Chronic kidney disease	4	64.58	1.101 (0.770 to 1.573)	0.597	1.192 (0.800 to 1.776)	0.387	1.626 (0.491 to 5.380)	0.510	4	1.101 (0.770 to 1.573)	0.597	0.827	0.800
Autoimmune	71	115.04	0.991 (0.884 to 1.111)	0.882	0.941 (0.796 to 1.113)	0.478	0.924 (0.758 to 1.127)	0.438	71	0.991 (0.884 to 1.111)	0.882	0.633	0.568
Celiac disease	32	525.66	0.960 (0.916 to 1.007)	0.095	0.949 (0.891 to 1.010)	0.097	0.973 (0.898 to 1.054)	0.505	32	0.960 (0.916 to 1.007)	0.095	0.936	0.940
Inflammatory bowel disease	178	101.09	1.003 (0.929 to 1.084)	0.932	0.914 (0.809 to 1.033)	0.150	0.892 (0.740 to 1.075)	0.233	178	1.003 (0.929 to 1.084)	0.932	0.576	0.567
Systemic lupus erythematosus	48	61.49	0.978 (0.929 to 1.030)	0.404	1.001 (0.926 to 1.081)	0.990	0.971 (0.866 to 1.088)	0.613	48	0.978 (0.929 to 1.030)	0.404	0.740	0.640
**Serum parameters**													
Serum calcium	7	304.27	**2.194 (1.063 to 4.530)**	**0.034**	2.103 (0.898 to 4.925)	0.087	2.052 (0.538 to 7.827)	0.341	7	**2.194 (1.063 to 4.530)**	**0.034**	0.995	0.991
Serum amylase	5	105.41	0.929 (0.794 to 1.086)	0.355	0.933 (0.790 to 1.103)	0.418	1.005 (0.589 to 1.717)	0.986	5	0.929 (0.794 to 1.086)	0.355	0.999	0.998
C-reactive protein	71	163.96	0.972 (0.781 to 1.210)	0.801	1.094 (0.794 to 1.508)	0.583	0.993 (0.714 to 1.380)	0.965	71	0.972 (0.781 to 1.210)	0.801	0.849	0.888
Testosterone	117	60.05	**0.435 (0.220 to 0.862)**	**0.017**	0.911 (0.293 to 2.830)	0.872	0.906 (0.279 to 2.940)	0.870	117	**0.435 (0.220 to 0.862)**	**0.017**	0.154	0.118
**Lipid metabolism**													
Triglycerides	85	130.38	**1.367 (1.075 to 1.737)**	**0.011**	**1.497 (1.054 to 2.126)**	**0.024**	**1.510 (1.015 to 2.245)**	**0.045**	85	**1.367 (1.075 to 1.737)**	**0.011**	0.196	0.224
HDL-C	117	128.43	0.859 (0.692 to 1.065)	0.165	0.831 (0.569 to 1.215)	0.340	0.779 (0.542 to 1.121)	0.181	117	0.859 (0.692 to 1.065)	0.165	0.370	0.516
LDL-C	65	172.44	0.915 (0.705 to 1.189)	0.508	0.706 (0.476 to 1.047)	0.083	0.854 (0.579 to 1.261)	0.431	65	0.915 (0.705 to 1.189)	0.508	0.058	0.046
Apolipoprotein A-I	92	124.37	0.951 (0.746 to 1.213)	0.688	0.893 (0.612 to 1.305)	0.560	0.907 (0.588 to 1.399)	0.660	92	0.951 (0.746 to 1.213)	0.688	0.301	0.359
Total cholesterol	73	139.04	0.948 (0.717 to 1.252)	0.704	0.785 (0.525 to 1.176)	0.240	0.692 (0.444 to 1.077)	0.107	70	0.933 (0.723 to 1.204)	0.185	0.041	0.050
**Glucose metabolism**													
Type 2 diabetes	131	870.63	1.093 (0.958 to 1.247)	0.187	1.140 (0.895 to 1.453)	0.289	1.037 (0.757 to 1.420)	0.823	131	1.093 (0.958 to 1.247)	0.187	0.609	0.606
Fasting glucose	84	125.65	1.585 (0.918 to 2.737)	0.098	**2.313 (1.034 to 5.174)**	**0.041**	2.194 (0.815 to 5.905)	0.124	84	1.585 (0.918 to 2.737)	0.098	0.224	0.298
HbA1c	87	102.51	1.492 (0.686 to 3.247)	0.313	1.502 (0.397 to 5.690)	0.549	2.916 (0.635 to 13.384)	0.172	87	1.492 (0.686 to 3.247)	0.313	0.484	0.504
Fasting insulin	42	50.67	0.819 (0.249 to 2.689)	0.742	0.429 (0.089 to 2.068)	0.291	0.092 (0.002 to 3.558)	0.208	42	0.819 (0.249 to 2.689)	0.742	0.101	0.100

**Obesity traits**													
Body mass index	414	55.44	1.106 (0.848 to 1.442)	0.458	0.946 (0.629 to 1.424)	0.791	0.543 (0.242 to 1.215)	0.138	414	1.106 (0.848 to 1.442)	0.458	0.246	0.287
Whole body fat mass	396	53.53	1.025 (0.772 to 1.362)	0.862	0.996 (0.658 to 1.508)	0.986	0.585 (0.240 to 1.427)	0.239	396	1.025 (0.772 to 1.362)	0.862	0.096	0.119
Waist circumference	70	47.59	1.302 (0.770 to 2.204)	0.325	1.385 (0.657 to 2.918)	0.392	1.420 (0.131 to 15.383)	0.774	70	1.302 (0.770 to 2.204)	0.325	0.071	0.074
Hip circumference	87	56.09	**0.581 (0.400 to 0.844)**	**0.004**	**0.467 (0.271 to 0.803)**	**0.006**	0.302 (0.072 to 1.262)	0.104	87	**0.581 (0.400 to 0.844)**	**0.004**	0.470	0.436
Waist-to-hip ratio	41	54.36	1.889 (0.996 to 3.583)	0.051	2.222 (0.933 to 5.288)	0.071	0.457 (0.020 to 10.206)	0.624	41	1.889 (0.996 to 3.583)	0.051	0.147	0.103

SNPs: single nucleotide polymorphisms; IVW: inverse variance weighted; WM: weighted median; MR-PRESSO: MR-pleiotropy residual sum and outlier; OR: odds ratio; CI: confidence interval; HDL-C: high-density lipoprotein cholesterol; LDL-C: low-density lipoprotein cholesterol; HbA1c: glycated hemoglobin.
